# Supplementary material for: Polyketide synthase gene clusters in the ericoid mycorrhizal fungus Oidiodendron maius: genome-wide prediction, expression profiling, and functional characterization of knockout mutants
Source: Front Plant Sci. 2026 Sep 16;17:1924430. doi: 10.3389/fpls.2026.1924430 (PMC13623903; doi:10.3389/fpls.2026.1924430)
Supplement: Supplementary file 1 [file SupplementaryFile1.zip › Supplementary Materials/Supplementary Folder 1.zip!/Oidma1_AssemblyScaffolds_Repeatmasked/knownclusterblast/cluster48/jgi.p_Oidma1_35074_mibig_hits.html]

| MIBiG Protein | Description | MIBiG Cluster | MiBiG Product | % ID | % Coverage | BLAST Score | E-value |
| --- | --- | --- | --- | --- | --- | --- | --- |
| AAK33073.1 | cytochrome\_P450 | BGC0001278 | Nivalenol\_/\_deoxynivalenol\_/\_3-acetyldeoxynivalenol\_/ | 32 | 99.430740038 | 263 | 6.4e-70 |
| AAK33083.1 | putative\_cytochrome\_P450 | BGC0001277 | Nivalenol\_/\_deoxynivalenol\_/\_3-acetyldeoxynivalenol\_/ | 32 | 99.8102466793 | 262 | 1.4e-69 |
| AAK53584.1 | trichodiene\_oxygenase | BGC0000931 | Trichothecene\_biosynthetic\_gene\_cluster | 32 | 99.8102466793 | 261 | 1.9e-69 |
| AAS89997.1 | CypA | BGC0000007 | Aflatoxin\_biosynthetic\_gene\_cluster | 36 | 96.2049335863 | 258 | 2e-68 |
| AAK53577.1 | trichodiene\_oxygenase | BGC0000930 | Trichothecene\_biosynthetic\_gene\_cluster | 31 | 99.8102466793 | 251 | 1.9e-66 |
| AIA58898.1 | putative\_cytochrome\_P450 | BGC0001141 | Brefeldin\_biosynthetic\_gene\_cluster | 32 | 97.7229601518 | 251 | 2.5e-66 |
| AIA58895.1 | putative\_cytochrome\_P450 | BGC0001141 | Brefeldin\_biosynthetic\_gene\_cluster | 31 | 97.9127134725 | 250 | 4.3e-66 |
| AAC49958.1 | cytochrome\_P450 | BGC0001279\_c2 | Deoxysambucinol\_/\_sambucinol\_/\_roridin\_E\_biosynthetic\_gene\_cluster | 31 | 99.430740038 | 245 | 1.8e-64 |
| BAV32149.1 | cytochrome\_P450\_monooxygenase | BGC0001373 | Sordarin\_biosynthetic\_gene\_cluster | 33 | 99.2409867173 | 233 | 4.1e-61 |
| EHA22197.1 | hypothetical\_protein | BGC0000170 | Yanuthone\_D\_biosynthetic\_gene\_cluster | 33 | 96.7741935484 | 232 | 9.2e-61 |
| Bcin12g06400.1 |  | BGC0000631 | Botrydial\_biosynthetic\_gene\_cluster | 29 | 92.220113852 | 210 | 3.8e-54 |
| BAV32152.1 | cytochrome\_P450\_monooxygenase | BGC0001373 | Sordarin\_biosynthetic\_gene\_cluster | 29 | 95.6356736243 | 208 | 1.4e-53 |
| AAS90045.1 | CypA | BGC0000009 | Aflatoxin\_biosynthetic\_gene\_cluster | 37 | 67.7419354839 | 207 | 5.4e-53 |
| Bcin12g06380.1 |  | BGC0000631 | Botrydial\_biosynthetic\_gene\_cluster | 28 | 93.1688804554 | 169 | 1.3e-41 |
| BAJ09784.1 | P450 | BGC0000146 | Solanapyrone\_biosynthetic\_gene\_cluster | 28 | 90.3225806452 | 168 | 2.1e-41 |
| XP\_002384857.1 | cytochrome\_P450,\_putative | BGC0001234\_c2 | 4,4'-piperazine-2,5-diyldimethyl-bis-phenol\_biosynthetic\_gene | 26 | 91.4611005693 | 123 | 1e-27 |
| ADI24958.2 | GsfF | BGC0000070 | Griseofulvin\_biosynthetic\_gene\_cluster | 26 | 93.3586337761 | 120 | 5.1e-27 |
| ACD39760.1 | cytochrome\_P450 | BGC0000077 | Hypothemycin\_biosynthetic\_gene\_cluster | 26 | 92.4098671727 | 118 | 3.3e-26 |
| ACD39751.1 | cytochrome\_P450 | BGC0000076 | Hypothemycin\_biosynthetic\_gene\_cluster | 26 | 92.4098671727 | 118 | 3.3e-26 |
| BAD29972.1 | P450\_monooxygenase\_1 | BGC0000676 | Aphidicolin\_biosynthetic\_gene\_cluster | 25 | 90.7020872865 | 115 | 1.6e-25 |
| BAD83682.1 | cytochrome\_P-450 | BGC0000012 | Alternapyrone\_biosynthetic\_gene\_cluster | 25 | 92.4098671727 | 111 | 3.1e-24 |
| BAE56591.1 |  | BGC0001123 | Aspirochlorine\_biosynthetic\_gene\_cluster | 24 | 90.5123339658 | 106 | 7.6e-23 |
| ADM34140.1 | P450 | BGC0001084 | Notoamide\_/\_stephacidin\_biosynthetic\_gene\_cluster | 25 | 91.6508538899 | 105 | 1.7e-22 |
| AAZ95016.1 | cytochrome\_P450\_monooxygenase | BGC0000048 | Dothistromin\_biosynthetic\_gene\_cluster | 24 | 99.430740038 | 105 | 2.2e-22 |
| ACH72905.1 | AflG | BGC0000011 | Aflatoxin/sterigmatocystin\_biosynthetic\_gene\_cluster | 25 | 100.0 | 103 | 8.4e-22 |
| AGC83578.1 | P450\_monooxygenase | BGC0000818 | Notoamide\_biosynthetic\_gene\_cluster | 25 | 91.4611005693 | 97 | 3.5e-20 |
| AAS90058.1 | AvnA | BGC0000009 | Aflatoxin\_biosynthetic\_gene\_cluster | 24 | 94.6869070209 | 97 | 4.6e-20 |
| AAC49196.1 | putative\_p450\_monooxygenase | BGC0000152 | Sterigmatocystin\_biosynthetic\_gene\_cluster | 24 | 90.3225806452 | 96 | 1e-19 |
| ACH72911.1 | AflV | BGC0000011 | Aflatoxin/sterigmatocystin\_biosynthetic\_gene\_cluster | 24 | 94.6869070209 | 94 | 5.1e-19 |
| ctg1\_orf000000 |  | BGC0000685 | Brassicicene\_C\_biosynthetic\_gene\_cluster | 37 | 21.2523719165 | 84 | 5.3e-16 |
| BAQ25465.1 | cytochrome\_P450 | BGC0001280 | Betaenone\_C\_/\_betaenone\_A\_biosynthetic\_gene\_cluster | 29 | 37.0018975332 | 78 | 2.2e-14 |
| ADZ25011.1 | cytochrome\_P450\_hydroxylase | BGC0000380 | Leupyrrin\_biosynthetic\_gene\_cluster | 29 | 35.6736242884 | 68 | 2.3e-11 |
| EAU32824.1 | predicted\_protein | BGC0000160 | Terreic\_acid\_biosynthetic\_gene\_cluster | 35 | 22.9601518027 | 67 | 6.7e-11 |
